# Study on the impact of biofilm formation by *Candida albicans* in recurrent vulvovaginal candidiasis on drug susceptibility

**DOI:** 10.3389/fcimb.2025.1663099

**Published:** 2025-09-25

**Authors:** Sainan Li, Zijia Shen, Suke Wang, Yongmei Peng, Wenjin Qi

**Affiliations:** ^1^ Department of Obstetrics and Gynecology, The First Affiliated Hospital of Kunming Medical University, Kunming, China; ^2^ Department of Obstetrics and Gynecology, The Third Affiliated Hospital of Guangzhou Medical University, Guangzhou, China; ^3^ Department of Gynecology, The Third Affiliated Hospital of Kunming Medical University, Kunming, China

**Keywords:** biofilm formation, drug resistance, *Candida albicans*, recurrent vulvovaginal candidiasis (RVVC), efflux pump genes (CDR1, CDR2)

## Abstract

**Background:**

Vulvovaginal candidiasis (VVC) and recurrent VVC (RVVC) are common fungal infections in women, often complicated by recurrence and treatment resistance. This study explores how *Candida albicans* biofilm formation influences antifungal susceptibility and resistance gene expression in clinical isolates.

**Methods:**

Clinical *Candida albicans* isolates were collected and identified. Biofilm formation ability was assessed using crystal violet staining and confocal laser scanning microscopy. Based on the strength of biofilm formation, isolates from VVC and RVVC patients were categorized into three groups: strong positive, moderate positive, and weak positive. The antifungal susceptibility of each strain to fluconazole, flucytosine, clotrimazole, and amphotericin B was determined using a modified broth microdilution method. The expression levels of the drug efflux pump genes CDR1, CDR2, and MDR1 were measured before and after biofilm formation in each group using RT-qPCR.

**Results:**

A higher proportion of strong biofilm-forming *Candida albicans* strains was of *Candida albicans* served in the RVVC group, whereas biofilm-negative strains were less common. In the VVC group, weak biofilm-formers produced significantly thinner biofilms than strong biofilm-formers in both VVC and RVVC (p<0.05). In RVVC weak biofilm-formers, amphotericin B exhibited higher MIC values than fluconazole and flucytosine (p<0.05), and across all RVVC subgroups, amphotericin B MICs were higher than those of clotrimazole (p<0.05). The MBEC of flucytosine was highest in the strongly positive VVC subgroup (p<0.05). In VVC, clotrimazole MBEC was lower in weak than in strong biofilm-formers (p<0.05). In RVVC, fluconazole MBEC was higher than flucytosine and clotrimazole in weak biofilm-formers, and also higher than amphotericin B in both weak and moderate biofilm-formers (p<0.05). Multivariate analysis further suggested that stronger biofilm-forming ability tended to increase the risk of RVVC independently of age, although this association did not reach statistical significance.

**Conclusions:**

Stronger biofilm-forming *Candida albicans* strains showed higher MBEC values. In RVVC, clotrimazole had the lowest MIC and MBEC, supporting its potential as a first-line treatment. Expression of **CDR1** and **CDR2** was highest in strong biofilm-forming strains, suggesting that biofilm formation promotes antifungal resistance through enhanced efflux gene expression, especially in RVVC.

## 1 Introduction

Vulvovaginal candidiasis (VVC) is a common lower genital tract infection in women caused by Candida species, with *Candida albicans* (CA) being the primary pathogen. Clinically, it is characterized by symptoms such as vulvovaginal itching, burning sensation, increased thick white curd-like or cottage cheese-like vaginal discharge, often accompanied by dyspareunia and dysuria (Fukazawa et al., 2019; Yano et al., 2019). Globally, approximately 75% of women experience at least one episode of VVC during their lifetime, 45% experience at least two episodes, and 5–8% suffer from four or more episodes annual (Sobel, 2016; Willems et al., 2020).

Microscopic examination of vaginal secretions from VVC patients often reveals budding yeast cells, single thin hyphae, or pseudohyphae with a segmented appearance. Candida species can be cultured for confirmation. When a patient experiences four or more clinically symptomatic VVC episodes within one year, it is defined as recurrent vulvovaginal candidiasis (RVVC) (Sobel, 2016; Ge et al., 2022).

*Candida albicans* is the most common causative agent of both VVC and RVVC and is considered an opportunistic pathogen. The onset of VVC and RVVC can be triggered by various factors, including diabetes, antibiotic use, elevated estrogen levels, sexual activity, and psychosocial stress. Antifungal agents are classified into six major categories: imidazoles, triazoles, pyrimidines, polyenes, echinocandins, and allylamines. Among them, azole antifungals are the most commonly used due to their efficacy and safety in treating vulvovaginal candidiasis (Mayer et al., 2013).

However, the improper use of antifungal agents in clinical practice, along with the overuse of antibiotics and immunosuppressants, has led to a gradual increase in drug resistance among C. albicans. The mechanisms of resistance include biofilm formation, alterations in drug targets, production of extracellular vesicles, overexpression of drug efflux pumps, changes in fungal cell wall composition, and calcium channel-related factors.

Biofilms are structured microbial communities that adhere to tissue surfaces and are enclosed within a self-produced extracellular matrix (ECM) composed of polysaccharides and extracellular DN (Rodríguez-Cerdeira et al., 2019). Biofilm formation is one of the major contributors to antifungal resistance. When planktonic microbes encounter and adhere reversibly to a surface during the initial “exploration” phase, they begin to form biofilms. Biofilms are highly organized microbial communities that exhibit reduced susceptibility to antifungal agents and enhanced dissemination of resistance, thereby offering a protective niche for opportunistic pathogen (Muzny and Schwebke, 2015).

In C. albicans, biofilm-induced resistance is mediated through several mechanisms, including limited drug penetration due to the ECM barrier, overexpression of efflux pumps, formation of persister cells, and phenotypic switching. Among its efflux systems, the overexpression of ATP-binding cassette (ABC) transporters plays a critical role in resistance. CDR1, CDR2, and MDR1 are among the most extensively studied efflux pump genes (Prasad et al., 2015). CDR1 was the first efflux pump gene identified in C. albicans. In clinical isolates, azole resistance is commonly mediated by the upregulation or overexpression of CDR1 and CDR2. These genes promote drug efflux, while upregulation of MDR1 not only enhances drug efflux but also decreases membrane permeability, thereby reducing intracellular drug concentration (Morschhäuser, 2002). Studies have shown that during the early stages of biofilm formation, CDR1, CDR2, and MDR1 are involved in the development of azole resistance (Mukherjee et al., 2003).

Currently, the main mechanisms of resistance in VVC and RVVC include alterations in drug targets, overexpression of efflux pumps, biofilm formation, changes in cell wall composition, calcium channel-related factors, and vesicle-mediated processes. *Candida albicans.*

Biofilms enhance the organism’s resistance to antifungal agents and host immune responses, allowing it to survive at antifungal concentrations up to 1,000 times higher than those needed to kill planktonic cells. Once formed, biofilms are difficult to eradicate, leading to persistent and recurrent infections that are hard to cure (Stepanovic et al., 2000).

In this study, we collected clinical strains from RVVC and VVC patients and identified *Candida albicans* among the isolates. We assessed the biofilm-forming ability of all C. albicans strains and evaluated their tolerance to antifungal drugs and host immune responses. Using a modified broth microdilution method, we measured the minimum inhibitory concentrations (MICs) of four commonly used antifungal agents—fluconazole, flucytosine, trimethoprim-sulfamethoxazole (SMZ-TMP), and amphotericin B—to investigate the impact of biofilm formation on drug resistance in C. albicans. This study also explores the mechanisms underlying biofilm-associated drug resistance in *Candida albicans*, aiming to provide new insights into the treatment of VVC and RVVC.

## 2 Methods and materials

### 2.1 Strain collection and identification

In this study, all strains were isolated from clinical specimens of patients diagnosed with VVC and RVVC. To ensure viability and purity, all isolates were subcultured on Sabouraud dextrose agar (Hopebio, China). Species identification was initially performed based on colony color using CHROMagar medium (Zhengzhou BioCell Biotechnology Co., China). In addition, the VITEK^®^ 2 system (bioMérieux, France) was used for further species confirmation. *Candida albicans* strains were selected for subsequent experiments.

### 2.2 Study population and inclusion criteria

Patients were recruited from the Department of Gynecology and Obstetrics, The First Affiliated Hospital of Kunming Medical University. Eligible participants were non-pregnant women of reproductive age who presented with typical symptoms of vulvovaginal candidiasis (VVC) or recurrent vulvovaginal candidiasis (RVVC), including vulvar or vaginal pruritus, burning sensation, increased thick white “cottage cheese-like” or curd-like vaginal discharge, dyspareunia, and/or dysuria. Written informed consent was obtained from all participants, and the study protocol was approved by the institutional ethics committee (Shao et al., 2022).

### 2.3 Diagnostic criteria

VVC was diagnosed based on (1) the presence of the above clinical symptoms or signs and (2) microscopic identification of budding yeast spores or pseudohyphae of *Candida albicans*. RVVC was defined as (1) four or more symptomatic VVC episodes within a 12-month period and (2) microscopic confirmation of budding yeast spores or pseudohyphae of *C. albicans* (Workowski et al., 2021).

### 2.4 Exclusion criteria

Patients were excluded if they had: (1) diabetes mellitus; (2) immune-related disorders or were receiving high-dose immunosuppressive therapy; (3) a history of long-term antibiotic use or prior exposure to immunosuppressants; (4) current high-dose estrogen therapy; (5) mixed vaginal infections; or (6) engaged in sexual intercourse, vaginal douching, or local medication within 24–48 hours before sample collection (Yao et al., 2016).

### 2.5 Isolate collection and grouping

According to the above criteria, a total of 151 C*. albicans* isolates were collected, including 58 strains from the VVC group and 93 strains from the RVVC group. Based on their biofilm-forming ability, the isolates were categorized into three groups: strong, moderate, and weak biofilm producers. From each group, seven strains were randomly selected for antifungal susceptibility testing against fluconazole, flucytosine, clotrimazole, and amphotericin B (Chirumbolo, 2014).

### 2.6 Gene expression analysis

The expression levels of efflux pump genes (CDR1, CDR2, and MDR1) were measured by RT-qPCR before and after biofilm formation in the selected isolates.

### 2.7 Crystal violet staining assay

To assess the biofilm-forming ability of *Candida albicans*, we employed a modified microtiter plate assay as previously reported. The fungal suspension was adjusted to a concentration of 5×10^6 CFU/mL using RPMI 1640 medium and thoroughly mixed. The working suspension was then inoculated into 96-well polystyrene cell culture plates (100 µL per well) (Labselect, China) and incubated at 37°C for 12 hours. Each strain was tested in triplicate. Wells containing only RPMI 1640 medium (Gibco, USA) served as blank controls. After incubation, the medium was discarded, and each well was gently washed twice with PBS to remove non-adherent cells. Subsequently, 100 µL of 0.1% crystal violet solution was added to each well and allowed to stain at room temperature for 20 minutes. Excess stain was removed by washing the biofilms twice with PBS. Finally, 100 µL of 95% ethanol was added to each well, and the plate was incubated at room temperature for 20 minutes. The optical density (OD) was then measured at 490 nm using a multimode plate reader (Bio-Rad Laboratories, Inc., USA).

All strains were classified into four categories based on their biofilm optical density (OD) values: negative (0), weakly positive (+), moderately positive (++), or strongly positive (+++). The cutoff OD value (ODc) for the microtiter plate assay was defined as the mean OD of the negative control plus three standard deviations. The specific classification criteria are shown in **Table 1**.

**Table 1:** Classification of biofilm OD values.

OD value	Results of evaluation
OD_490nm_ ≤ ODc	Negative
ODc< OD_490nm_ ≤ 2 ×ODc	Weakly positive
2 ×ODc< OD_490nm_ ≤ 4 ×ODc	Moderately positive
4 ×ODc< OD_490nm_	Strongly positive

### 2.8 Cell wall staining and measurement

Biofilm thickness was measured using confocal laser scanning microscopy (CLSM). Sterile microscope coverslips were placed at the bottom of 6-well plates, and 2 mL of fungal suspension was added to each well. The plates were incubated at 37°C for 24 hours. After incubation, the coverslips were gently removed and washed twice with PBS. They were then incubated with FITC in the dark at 4°C for 30 minutes, followed by another PBS wash.

Next, 10 μL of antifade mounting medium was dropped onto a glass slide, and the coverslip with the adherent cells was placed on top. A laser with excitation wavelengths of 405 nm and 488 nm was used as the light source. From the outer surface of the biofilm, Z-stack scanning was performed at 1 μm intervals. The number of layers from the first appearance of fluorescence to its final disappearance was recorded, and the thickness of the *Candida albicans* biofilm was calculated accordingly.

### 2.9 Antifungal agents

Fluconazole, flucytosine, clotrimazole, and amphotericin B were obtained from the National Institutes for Food and Drug Control (China), while 5-fluorocytosine was purchased from Beijing Solarbio Science & Technology Co., Ltd.

### 2.10 Antifungal susceptibility testing

A modified broth microdilution method, based on the Clinical and Laboratory Standards Institute (CLSI) M27-A3 guidelines for antifungal susceptibility testing of yeasts, was used to determine the susceptibility of test strains to four antifungal agents—fluconazole, flucytosine, clotrimazole, and amphotericin B—before and after biofilm formation. The minimum inhibitory concentration (MIC) and minimum biofilm clearance concentration (MBEC) were determined. *Candida albicans* was cultured in RPMI 1640 medium, and results were assessed using the Alamar Blue indicator. Candida parapsilosis ATCC 22019 and Candida krusei ATCC 6258, obtained from the American Type Culture Collection (ATCC), were used as quality control strains.

Fluconazole, being highly soluble in water, was weighed using an electronic balance and dissolved in sterile distilled water to prepare a stock solution at 5120 µg/mL. Flucytosine, clotrimazole, and amphotericin B, which have poor water solubility, were initially dissolved in 100 µL dimethyl sulfoxide (DMSO) until fully dissolved and then diluted with sterile distilled water to a final concentration of 1280 µg/mL (with the final DMSO concentration not exceeding 2%). The stock solutions were sterilized using a 0.22 μm filter, aliquoted into sterile 1.5 mL centrifuge tubes, and stored at −20°C for later use. Working solutions were prepared by diluting the stock solutions with RPMI 1640 medium. Final working concentrations were: fluconazole diluted from 5120 µg/mL to 128 µg/mL; clotrimazole from 1280 µg/mL to 64 µg/mL; and flucytosine and amphotericin B from 1280 µg/mL to 32 µg/mL.

The fungal suspension was adjusted to 5×10³ CFU/mL using RPMI 1640 medium. In a 96-well microdilution plate, 100 µL of RPMI 1640 medium (Gibco, USA) was added to wells in columns 1 to 11, and 200 µL of RPMI 1640 medium was added to column 12 as the negative control. Column 11 contained only fungal inoculum and served as the positive control. Antifungal agents were added to wells in columns 2 to 10 using two-fold serial dilutions. The concentration ranges were as follows: fluconazole, 0.125–64 µg/mL; clotrimazole, 0.06–32 µg/mL; and all other antifungal agents, 0.0313–16 µg/mL.

To determine MICs, 20 µL of freshly prepared Alamar Blue reagent was added to each well and mixed thoroughly. The plates were incubated at 37°C for 24 hours. MICs were recorded as the lowest concentration of the drug at which no color change (from blue to red) occurred. The results were considered valid only if the negative control wells remained blue, the positive control wells turned red, and the MICs of quality control strains fell within the established acceptable range. *Candida albicans* ATCC 90028, obtained from the American Type Culture Collection, was used as a quality control strain.

Following biofilm formation, MBECs were determined using the same procedure. If fungal growth was observed in all wells at the tested antifungal concentrations, higher concentrations were tested. Conversely, if no growth was observed in any well, testing was repeated starting from ½ MIC concentration for that strain and antifungal agent.

Based on the MIC interpretive criteria established in CLSI M27-A3, all isolates were categorized as susceptible (S), intermediate (I), or resistant (R). The breakpoint values for fluconazole, flucytosine, clotrimazole, and amphotericin B are shown in **Table 2**.

**Table 2:** Breakpoint values of antifungal agents.

Antifungal	S	I	R
Fluconazole Flucytosine	≤8μg/mL ≤4μg/mL	16-32μg/mL 8-16μg/mL	≥64μg/mL ≥32μg/mL
Clotrimazole	≤1μg/mL	2μg/mL	≥4μg/mL
Amphotericin B	≤1μg/mL	2μg/mL	≥4μg/mL

### 2.11 Quantitative real-time polymerase chain reaction analysis

Total RNA from the lavage fluid was extracted using a column-based yeast total RNA extraction and purification kit (Sangon Biotech, China) following the manufacturer’s instructions. RNA purity and concentration were assessed by measuring OD260 and OD280 using a spectrophotometer. Reverse transcription of mRNA was performed using the TransScript^®^ Uni All-in-One First-Strand cDNA Synthesis SuperMix for qPCR (One-Step gDNA Removal) kit (TransGen Biotech, China).

The resulting cDNA was diluted 5–20 fold with 0.05 mL of RNase/DNase-free ddH_2_O and used for qRT-PCR with PerfectStart^®^ Green qPCR SuperMix (TransGen Biotech, China). The primers used are listed in **Table 3**. Relative gene expression levels were calculated using the 2^-ΔΔCt method. The ΔCt value was calculated as follows: ΔCt [test gene]=Ct [test gene] – Ct [18S rRNA].

**Table 3:** Primer gene sequences.

Primer	Oligonucleotide sequence
18SrRNA	F:5′-TCTTTCTTGATTTTGTGGGTGG-3′
	R: 5′-TCGATAGTCCCTCTAAGAAGTG-3′
CDR1	F: 5′-TGTGTACTATCCATCAACCATCAGC-3′
	R: 5′-CACCAAAATAAGCCGTTCTACCA-3′
CDR2	F: 5′-TTTTCGTTCCATTCACGACA-3′
	R: 5′-CCAGCAATAAATGCAAACCA-3′
MDR1	F: 5′-TCAGCGGGTTCTTTGTTGTATG-3′
	R:5′-GATAATGTTTAGCAAGCCGAGGA-3′

## 3 Statistical analysis

All data were analyzed using SPSS version 26.0. Each experiment was repeated at least three times, and results were expressed as mean ± standard deviation (SD). Differences between two groups were compared using the t-test, while comparisons among multiple groups were conducted using one-way analysis of variance (ANOVA).

Spearman correlation analysis was used to evaluate the relationships between the two biofilm quantification methods, between MIC and MBEC values, between MBEC and biofilm formation ability, and between the increase in drug resistance before and after biofilm formation and biofilm-forming capacity.

For data not conforming to a normal distribution, results were presented as median and interquartile range (IQR), and intergroup differences were assessed using the Wilcoxon signed-rank test. A p-value less than 0.05 was considered statistically significant, while a p-value greater than 0.05 indicated no significant difference.

## 4 Results

### 4.1 Biofilm-forming ability of *Candida albicans* in VVC and RVVC

The distribution and number of strains in each group are shown in **Table 4**. There was a statistically significant difference in the composition ratio of biofilm-forming ability between the VVC and RVVC groups (χ²=10.046, p=0.018). Specifically, the proportions of strongly positive and negative biofilm-forming strains differed significantly between the two groups (p<0.05), with the RVVC group exhibiting a higher proportion of strong biofilm producers and a significantly lower proportion of non-biofilm-forming strains compared to the VVC group. However, there were no statistically significant differences between the two groups in the proportions of moderately and weakly positive biofilm-forming strains (p > 0.05).

**Table 4:** Biofilm-forming ability of *Candida albicans* in VVC and RVVC measured by crystal violet staining.

Group	Total [n (%)]	VVC [n (%)]	RVVC [n (%)]	χ2 test	
				χ2	p
strong	61 (40.4%)	16 (27.6%)	45 (48.4%)*	10.046	0.018
moderate	34 (22.5%)	15 (25.9%)	19 (20.4%)		
weak	45 (29.8%)	19 (32.8%)	26 (28.0%)		
negative	11 (7.3%)	8 (13.8%)	3 (3.2%)^#^		

*p < 0.05, compared with the VVC group, the proportion of strong biofilm-forming strains was higher, with a statistically significant difference;

#*p* < 0.05, compared with the VVC group, the proportion of biofilm-negative strains was lower, with a statistically significant difference.

### 4.2 Biofilm thickness after formation in VVC and RVVC groups measured by confocal microscopy

**Figure 1:**
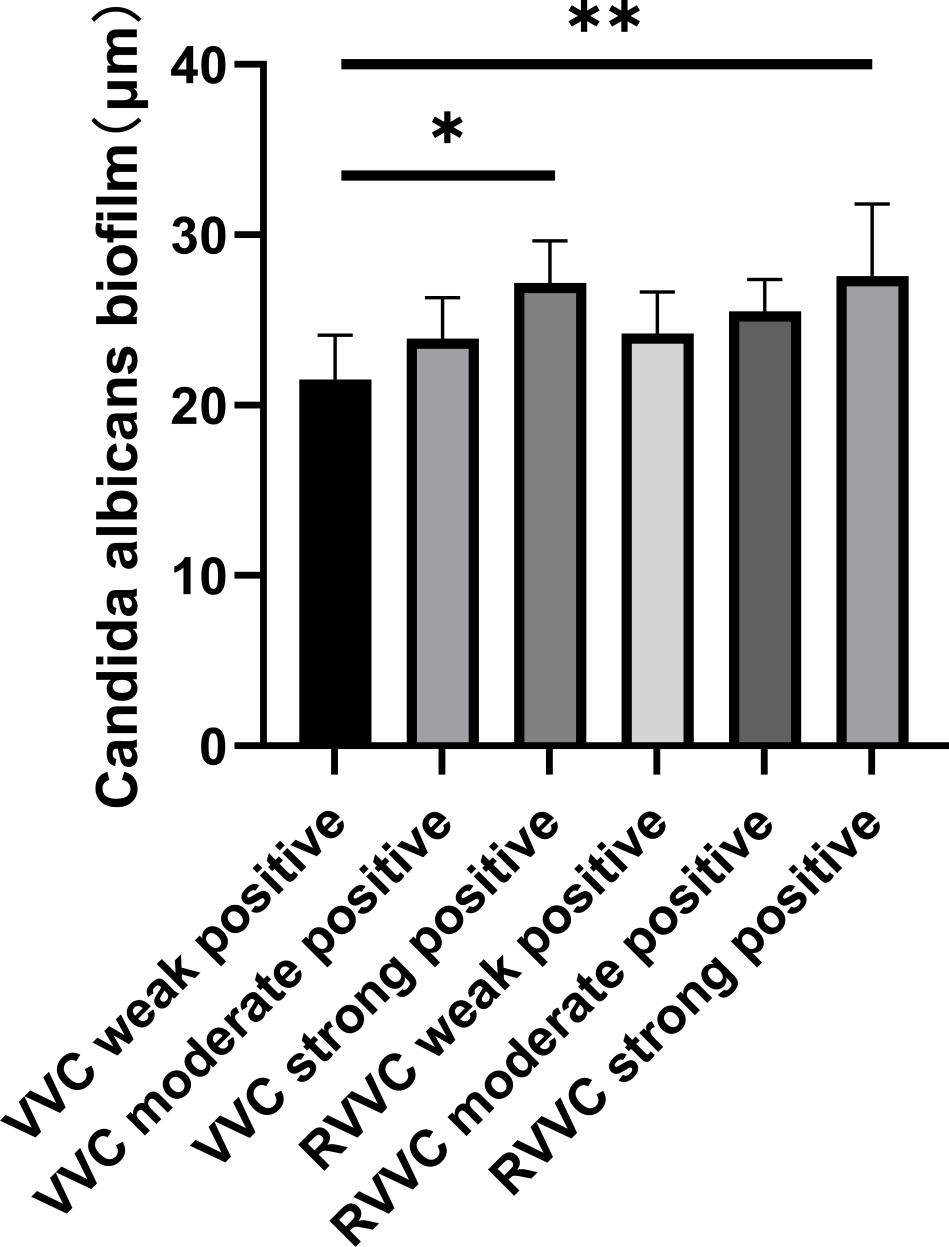
Biofilm thickness measured by confocal laser scanning microscopy. Representative confocal images of biofilms formed by *Candida albicans* isolates from patients with vulvovaginal candidiasis (VVC) and recurrent VVC (RVVC). Biofilms were stained with the LIVE/DEAD BacLight viability kit: green fluorescence indicates viable yeast cells with intact membranes (SYTO 9), while red fluorescence indicates dead or membrane-compromised cells (propidium iodide). Images were captured at ×400 magnification using a confocal laser scanning microscope. Z-stack reconstruction was applied to evaluate biofilm architecture and thickness. Compared with VVC isolates, RVVC isolates exhibited denser, multilayered biofilm structures with increased biomass and higher proportions of viable cells, suggesting stronger biofilm-forming capacity that may contribute to recurrent infection.

The biofilm thickness of *Candida albicans* strains with different biofilm-forming abilities in the VVC and RVVC groups, as measured by confocal laser scanning microscopy, is shown in **Figure 1**.

The biofilm thickness in the VVC weakly positive group was significantly lower than that in the VVC strongly positive group, the RVVC moderately positive group, and the RVVC strongly positive group (p<0.05). Similarly, the VVC moderately positive group showed significantly lower biofilm thickness compared to the VVC strongly positive and RVVC strongly positive groups (p<0.05).

In the RVVC group, the biofilm thickness of the weakly positive subgroup was significantly lower than that of the strongly positive subgroup (p<0.05).

**Figure 2:**
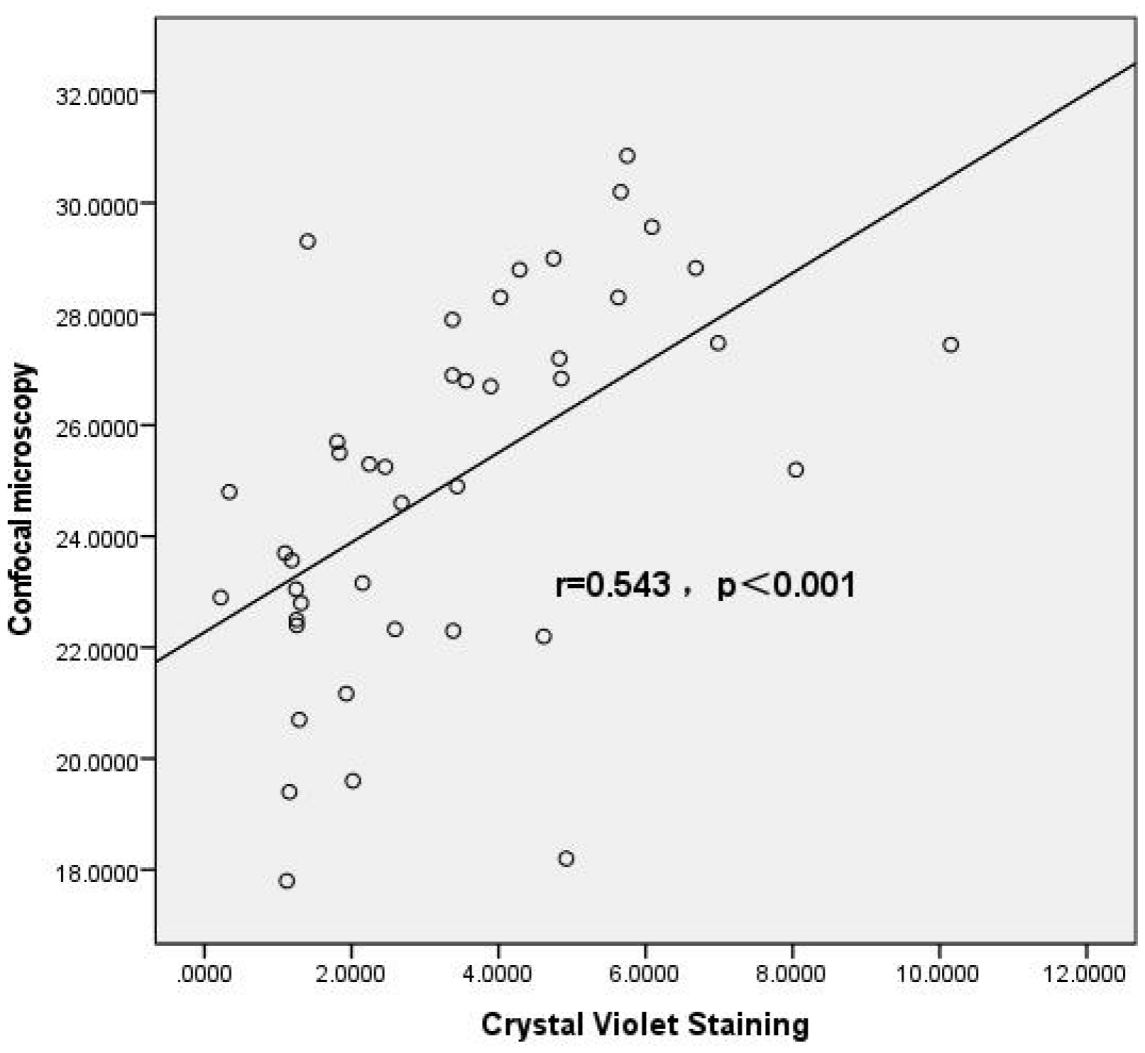
Correlation analysis between crystal violet staining and confocal microscopy methods. Scatter plot illustrating the correlation between biofilm biomass quantified by crystal violet (CV) staining and biofilm thickness determined by confocal laser scanning microscopy (CLSM) in *Candida albicans* isolates. Each point represents an individual clinical isolate. A significant positive correlation was observed between the two methods (Spearman’s correlation coefficient r=0.543, *p*<0.001), indicating that isolates forming higher biomass in CV staining assays also exhibited increased biofilm thickness under CLSM observation. The regression line demonstrates the trend between the two variables. This result validates the use of CV staining as a reliable and convenient method for estimating biofilm formation, consistent with structural measurements obtained by CLSM.

The results of Spearman correlation analysis showed that the correlation coefficient between the crystal violet staining method (OD490nm) and the confocal microscopy method (biofilm thickness) for evaluating biofilm-forming ability was r=0.595, p<0.001. A positive correlation was observed between the two methods, indicating good consistency in the results (**Figure 2**).

### 4.3 Effect of biofilm formation on antifungal susceptibility of *Candida albicans* antifungal susceptibility testing before biofilm formation

Susceptibility tests for fluconazole, flucytosine, clotrimazole, and amphotericin B were performed on strains isolated from patients with VVC and RVVC. The results are shown in **Table 5**.

**Table 5:** Antifungal susceptibility of *Candida albicans* isolated from VVC and RVVC to four antifungal agents.

Antifungal	VVC [n (%)]			RVVC [n (%)]		
	S	I	R	S	I	R
Fluconazole	19(90.0%)	2 (10%)	0	21(100%)	0	0
Flucytosine	17(81.0%)	4(19.0%)	0	19(90%)	2 (10%)	0
Clotrimazole	20(95.2%)	1 (4.8%)	0	21(100%)	0	0
Amphotericin B	10(47.6%)	4(19.1%)	7(33.3%)	3(14.3%)	6(28.6%)	12(57.1%)

When comparing the 24-hour MIC values of fluconazole, flucytosine, clotrimazole, and amphotericin B among VVC and RVVC groups with different biofilm-forming abilities, it was found that in the RVVC weakly positive group, there was a statistically significant difference in MICs between amphotericin B and the other three antifungal agents (p=0.002). Amphotericin B exhibited a higher MIC against C. albicans compared to fluconazole, flucytosine, and clotrimazole.

In the RVVC moderately positive group, the MIC of clotrimazole was significantly lower than that of amphotericin B (p=0.024). Similarly, in the RVVC strongly positive group, clotrimazole showed a significantly lower MIC compared to amphotericin B (p<0.05) (**Tables 5**, **6**).

**Table 6:** 24-hour MIC (μg/mL) of four antifungal agents against *Candida albicans* from VVC and RVVC.

Group	Fluconazole	Flucytosine	Clotrimazole	Amphotericin B
VVC-weak	1 (0.13,4)	2 (1,8)	0.0625 (0.0625,0.5)	2 (0.31,4)
VVC moderate	5 (0.25,8)	0.5 (0.25,1)	0.06 (0.06,0.06)	0.5 (0.31,1)
VVC strong	2 (1,4)	4 (2,16)	0.06 (0.06,0.06)	4 (2,8)
RVVC weak	5 (0.5,2)*	1 (0.5,2)*	0.06 (0.06,0.06)*	8 (2,16)*
RVVC moderate	2 (0.25,2)	4 (0.5,4)	0.06 (0.06,0.06)#	4 (2,8)#
RVVC strong	1 (0.25,8)	2 (0.5,4)	0.06 (0.06,0.06)**	2 (2,16)**

*Note: In the RVVC weakly positive group, the MIC of amphotericin B was significantly different from those of the other three antifungal agents (*p* < 0.05);

#In the RVVC moderately positive group, the MIC of amphotericin B was significantly different from that of clotrimazole (*p* < 0.05);

**In the RVVC strongly positive group, the MIC of amphotericin B was significantly different from that of clotrimazole (*p* < 0.05).

### 4.4 Antifungal susceptibility after biofilm formation: MBEC values of fluconazole and clotrimazole

After biofilm formation, the MBEC values of fluconazole, flucytosine, clotrimazole, and amphotericin B were compared among VVC and RVVC groups with different levels of biofilm-forming ability. The results showed that there were statistically significant differences in MBEC values among the antifungal agents (H=39.218, p<0.001) as well as among the biofilm formation groups (H=26.143, p<0.001).

Pairwise comparisons revealed that the MBEC of flucytosine in the VVC strongly positive group was significantly higher than that in the other five groups (p=0.01), including the VVC weakly and moderately positive groups, and the RVVC weakly, moderately, and strongly positive groups.

For clotrimazole, the MBEC in the VVC strongly positive group was significantly higher than in the weakly positive group (p=0.02).

In the RVVC weakly positive group, the MBEC of fluconazole was significantly higher than those of the other three antifungal agents—flucytosine, clotrimazole, and amphotericin B (p=0.011).

In the RVVC moderately positive group, the MBEC of fluconazole was significantly higher than that of amphotericin B (p=0.015) (**Tables 7**, **8**).

**Table 7:** Composition ratios of susceptible and resistant *Candida albicans* strains from VVC and RVVC after biofilm formation.

Drug name	VVC [n (%)]			RVVC [n (%)]		
	S	I	R	S	I	R
Fluconazole	5 (23.8%)	3 (14.3%)	13 (61.9%)	1 (4.8%)	3 (14.3%)	17 (80.9%)
Flucytosine	5 (23.8%)	4 (19.0%)	11 (52.2%)	1 (4.8%)	3 (14.3%)	17 (80.9%)
Clotrimazole	7 (33.3%)	2 (9.5%)	12 (57.2%)	0	2 (9.5%)	19 (90.5%)
Amphotericin B	6 (28.6%)	1 (4.8%)	14(66.6%)	7 (33.3%)	1 (4.8%)	13(61.9%)

**Table 8:** The 24-hour MBEC of four drugs to *Candida albicans* in VVC and RVVC.

Grouping	Fluconazole	Flucytosine	Clotrimazole	Amphotericin B
VVC Weakly Positive	31 (8,250)	16 (8,32)^*^	2 (0.0625,4)^#^	4 (0.5,16)
VVC Moderately Positive	250 (2,3000)	8 (1,128)^*^	32 (0.0625,600)	2 (1,16)
VVC Strongly Positive	2000 (32,4000)	1000 (512,2000)^*^	1000 (8,1500)^#^	32 (8,60)
RVVC Weakly Positive	2000 (31,2000)^+^	125 (8,250)^*+^	125 (2,1000)^+^	4 (0.5,30)^+^
RVVC Moderately Positive	1000 (250,3000)^**^	375 (250,600)^*^	600 (32,1500)	5 (0.5,60)^**^
RVVC Strongly Positive	500 (125,2000)	375 (125,375)^*^	125 (30,1000)	4 (1,16)

*The MBEC of flucytosine in the VVC strongly positive group was significantly different from that in all other groups (*p* < 0.05);

**#**There was a significant difference in the MBEC of clotrimazole between the VVC strongly positive and weakly positive groups (*p* < 0.05);

+In the RVVC weakly positive group, the MBEC of clotrimazole was significantly different from those of the other antifungal agents (*p* < 0.05);

**In the RVVC moderately positive group, the MBEC values of flucytosine and amphotericin B were significantly different (*p* < 0.05).

### 4.5 Comparison of drug resistance in *Candida albicans* before and after biofilm formation

The distributions of susceptible (S), intermediate (I), and resistant (R) *Candida albicans* strains to four antifungal agents before and after biofilm formation were statistically analyzed, as shown in **Table 9**.

**Table 9:** Analysis of susceptible and resistant *Candida albicans* strain proportions before and after biofilm formation.

Drug name	VVC		RVVC	
	χ2 value	p	χ2 value	p
Fluconazole	23.585	0.000*	43.653	0.000*
Flucytosine	19.026	0.000*	38.823	0.000*
Clotrimazole	20.388	0.000*	50.176	0.000*
Amphotericin B	4.908	0.092	4.995	0.086

*The distribution of susceptible (S) and resistant (R) strains before and after biofilm formation showed a statistically significant difference (*p* < 0.05).

For fluconazole, flucytosine, and clotrimazole, the number of resistant (R) strains increased significantly and susceptible (S) strains decreased in both VVC and RVVC groups.

In both the VVC and RVVC groups, the proportions of S and R strains for fluconazole, flucytosine, and clotrimazole showed significant differences before and after biofilm formation (p<0.05). After biofilm formation, the number of R strains increased significantly, while the number of S strains decreased in both VVC and RVVC groups. However, the distribution of I strains showed no significant change (p > 0.05).

For amphotericin B, there were no significant differences in the proportions of S, I, and R strains before and after biofilm formation (p > 0.05).

To assess the impact of biofilm formation on antifungal susceptibility, the fold increase between MBEC and MIC values was calculated and expressed as log_2_-transformed dilution steps (i.e., changes in two-fold dilution levels). Among the 42 *Candida albicans* strains tested, after biofilm formation, the average increase in resistance was equivalent to 8.30, 6.82, 11.25, and 2.31 two-fold dilutions for fluconazole, flucytosine, clotrimazole, and amphotericin B, respectively. This indicates that biofilm formation conferred the highest increase in resistance to clotrimazole and the lowest to amphotericin B.

Using the Wilcoxon signed-rank test (z=–10.179, p<0.001), a statistically significant difference was observed in the MIC values of C. albicans before and after biofilm formation, indicating that biofilm formation leads to increased antifungal resistance.

**Figure 3:**
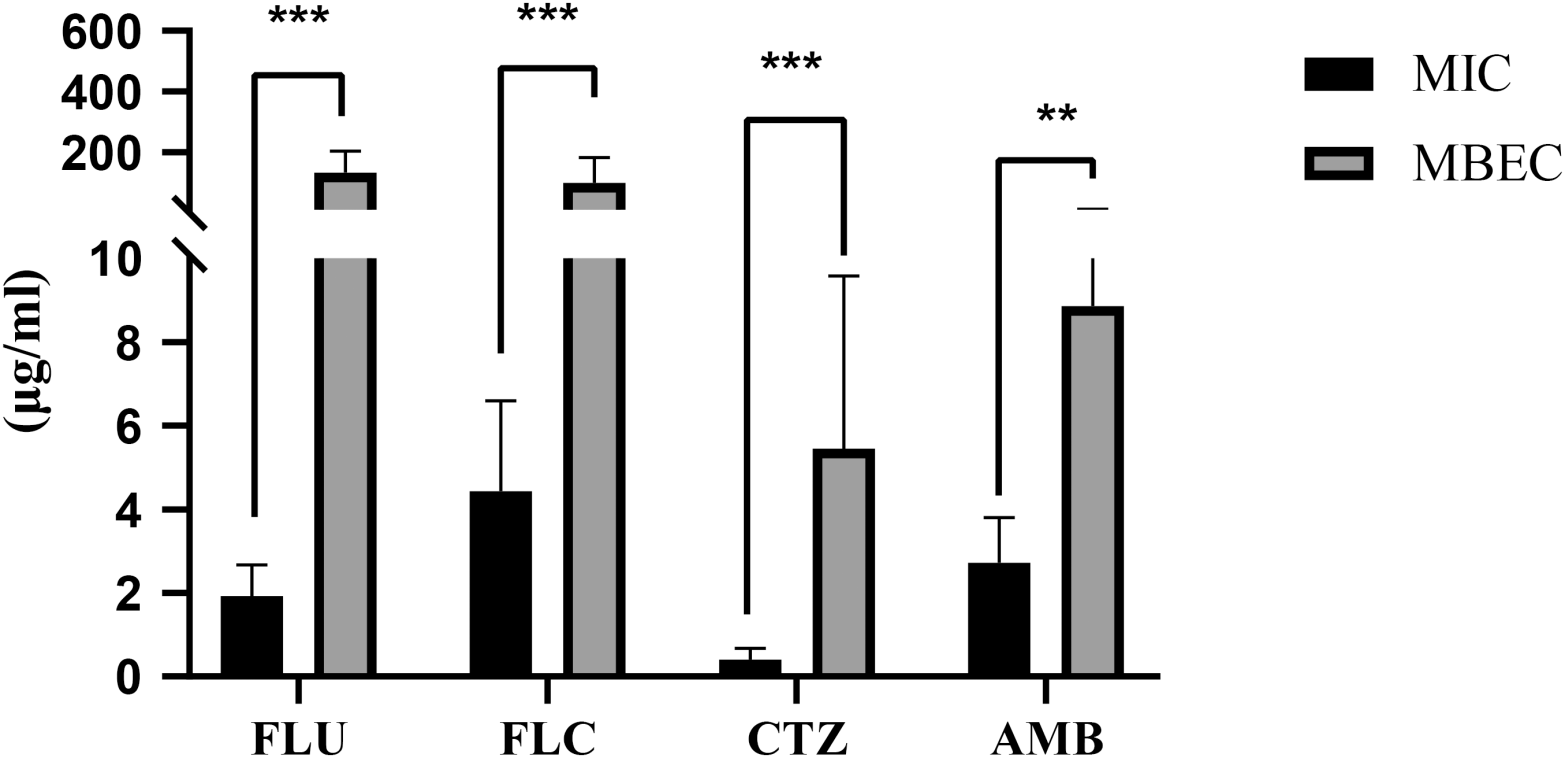
MIC and MBEC Values of Fluconazole, Flucytosine, Clotrimazole, and Amphotericin (B) Bar graphs illustrate the minimum inhibitory concentrations (MICs) and minimum biofilm eradication concentrations (MBECs) of four commonly used antifungal agents: fluconazole, flucytosine, clotrimazole, and amphotericin (B) MIC values represent the lowest concentration of antifungal drug required to inhibit planktonic growth, whereas MBEC values indicate the concentration required to eradicate established biofilms. Data are presented as mean ± standard deviation (SD) from three independent experiments. Statistical analysis was performed using the Kruskal–Wallis test followed by *post-hoc* comparisons. A highly significant difference was observed across the antifungal groups (*p*<0.001). Double asterisks (**) denote comparisons where *p*<0.01. Overall, amphotericin B exhibited higher MIC and MBEC values compared to azoles (fluconazole, clotrimazole), indicating reduced efficacy against biofilm-associated cells, while flucytosine showed variable but generally lower activity. These results highlight differences in antifungal susceptibility between planktonic and biofilm-associated *C*. *albicans* cells.

Further analysis (**Figure 3**) showed statistically significant differences in MIC values before and after biofilm formation for all four antifungal agents (p<0.05). These results confirm that biofilm formation significantly enhances C. albicans resistance to fluconazole, flucytosine, clotrimazole, and amphotericin B.

### 4.6 Effect of biofilm formation on the expression of CDR1, CDR2, and MDR1 genes

**Figure 4:**
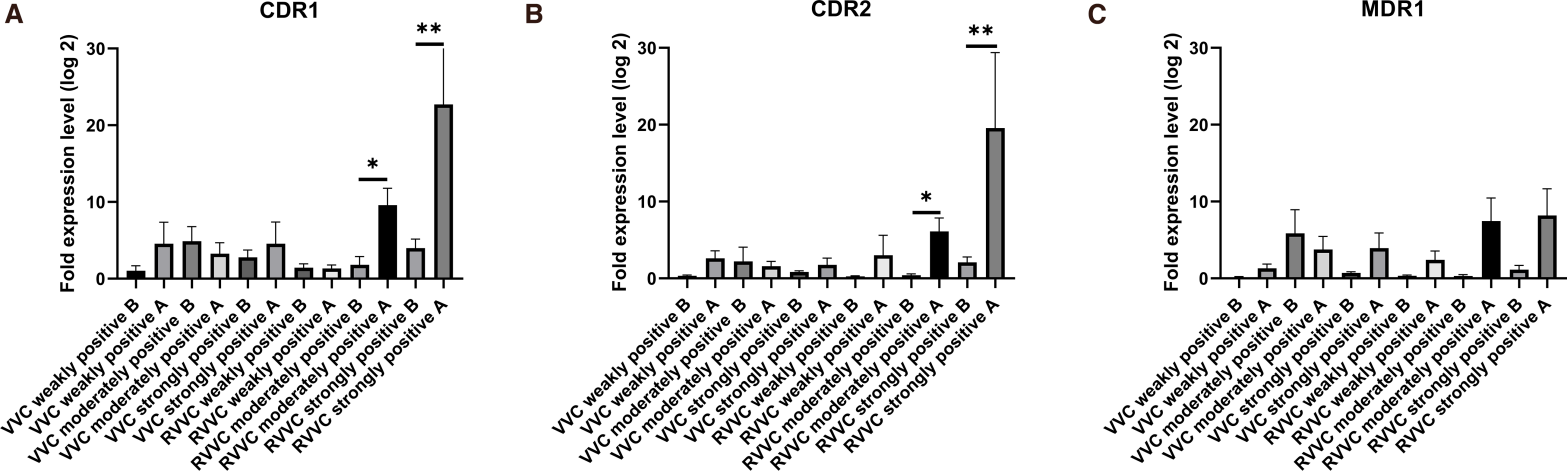
Fold changes in CDR1, CDR2, and MDR1 expression levels in *Candida albicans* before and after biofilm formation. **(A)** Relative mRNA expression of CDR1 in vaginal isolates from patients with vulvovaginal candidiasis (VVC) and recurrent VVC (RVVC). **(B)** Relative mRNA expression of CDR2 in isolates from the same groups. **(C)** Relative mRNA expression of MDR1 in isolates from VVC and RVVC. Gene expression levels were quantified using quantitative real-time PCR (qRT-PCR) and normalized to ACT1 as the housekeeping gene. Each bar represents the mean ± standard deviation (SD) from at least three independent biological replicates. Statistical significance was assessed using the Mann–Whitney U test; *p*<0.05 was considered significant. Increased expression of efflux pump–related genes (CDR1, CDR2, MDR1) in the RVVC group suggests a potential association with enhanced antifungal resistance and recurrent infection.

The expression patterns of CDR1, CDR2, and MDR1 showed similar overall trends (**Figure 4**). Both CDR1 and CDR2 expression levels were significantly upregulated after biofilm formation (p<0.05).

For CDR1, gene expression in the RVVC strongly positive strains was significantly higher than in all other groups (p<0.05), except for the RVVC moderately positive group, with which it showed no significant difference (p > 0.05). No significant differences were observed among the remaining groups.

Regarding CDR2, expression in the RVVC strongly positive group was also significantly higher than in all other groups (p<0.05), but not significantly different from the RVVC moderately positive group (p > 0.05). No significant differences were found among the other groups (p > 0.05).

For MDR1 (**Figure 4**), there were no statistically significant differences in expression among the groups, indicating that biofilm formation did not affect its expression (p > 0.05).

### 4.7 Multivariate logistic regression analysis

To further explore the independent association between clinical variables and RVVC, a multivariate logistic regression model was constructed with RVVC (yes/no) as the dependent variable, and age and biofilm-forming ability as independent variables. The results showed that biofilm-forming ability was positively associated with RVVC risk (OR=1.46, 95% CI: 0.97–2.19, p=0.070), while age was not significantly related to RVVC (OR=1.02, 95% CI: 0.98–1.07, p=0.320).

## 5 Discussion

*Candida albicans* exists in two developmental states: planktonic and biofilm. A biofilm is defined as a microbial community that adheres to a surface and is surrounded by an extracellular matrix (ECM) (Muzny and Schwebke, 2015; Rodríguez-Cerdeira et al., 2019). When planktonic cells encounter a surface that may provide nutrients or other advantages, they reversibly attach to it and begin forming a biofilm. Biofilm formation involves four stages: adhesion, proliferation, maturation, and dispersion (Silva et al., 2009; Lohse et al., 2018). Adhesion occurs during the first 0–11 hours and is a critical step in biofilm development. C. albicans adheres to the vaginal mucosa, and the strength of adhesion is positively correlated with its pathogenicity and drug resistance.

During the proliferation phase (12–13 hours), hyphae form, and extracellular matrix is produced. Hyphae serve as the structural backbone of the biofilm, supporting the attachment of yeast, other hyphae, and pseudohyphae. Between 31–72 hours, the biofilm matures as hyphae develop and the ECM becomes more established, resulting in increased resistance (Gulati and Nobile, 2016). After 72 hours, yeast cells are dispersed from the mature biofilm and colonize new surfaces, leading to recurrent infection (Rodríguez-Cerdeira et al., 2019).

In this study, a biofilm formation assay was performed, and 92.7% of C. albicans isolates were capable of forming biofilms: 40.4% were strongly positive, 22.5% moderately positive, and 29.8% weakly positive. The RVVC group showed a significantly higher proportion of strong biofilm producers than the VVC group, suggesting that biofilm formation may contribute to the recurrence of RVVC. Fluorescent staining and confocal laser scanning microscopy (CLSM) were used to visualize and measure biofilm thickness. Results showed that biofilm thickness was lower in the VVC weakly positive group compared to the VVC strongly positive, RVVC moderately positive, and RVVC strongly positive groups; the VVC moderately positive group also had lower biofilm thickness than the VVC strongly positive and RVVC strongly positive groups; and the RVVC weakly positive group had lower thickness than the RVVC strongly positive group. In general, biofilms from RVVC isolates were thicker than those from VVC isolates. Thicker biofilms correlated with higher OD values in crystal violet staining, indicating consistency between the two evaluation methods.

C. albicans isolates from VVC and RVVC were categorized into strong, moderate, and weak biofilm-forming groups. MICs and MBECs for fluconazole, flucytosine, clotrimazole, and amphotericin B were determined using a modified broth microdilution method. Previous studies have shown that biofilms contribute to fluconazole resistance in RVV (Pan et al., 2023). Our findings indicate that biofilm formation significantly decreased the number of susceptible strains and increased the number of resistant strains for fluconazole, flucytosine, and clotrimazole. In contrast, the susceptibility profile for amphotericin B remained largely unchanged; biofilm formation conferred the highest resistance increase to clotrimazole and the lowest to amphotericin B, suggesting a strong correlation between biofilm formation and azole resistance (Gerges et al., 2023). Amphotericin B, a polyene antifungal that disrupts fungal cell membranes, was less affected by biofilm formation. Previous studies have reported that amphotericin B can inhibit or disrupt biofilms either alone or in combination therap (Maiolo et al., 2014), which warrants further investigation into its mechanism of action against C. albicans biofilms.

We also analyzed the correlations between MIC and MBEC, MBEC and biofilm-forming ability, and found that biofilm formation affected resistance to the four antifungal agents inconsistently. MBEC values for flucytosine and amphotericin B increased with stronger biofilm formation, suggesting limited effectiveness of these drugs against biofilm-associated infections. Strains resistant to fluconazole in the planktonic state generally remained resistant in the biofilm state. No significant correlation was found between MIC and MBEC for clotrimazole, suggesting it may be the most effective drug against C. albicans biofilms, consistent with previous finding (Tits et al., 2020).

The mechanisms of biofilm-associated drug resistance in *C. albicans* include physical barriers, efflux pump activity, persister cell formation, and phenotypic changes. Azole resistance is mainly related to the overexpression of efflux pumps, especially the ABC transporters CDR1 and CDR2, and the MFS transporter MDR1 (Lewis, 2012; Tong et al., 2016; Cavalheiro and Teixeira, 2018). ABC transporters use ATP to actively expel drugs, while MFS transporters rely on proton gradients (Maras et al., 2021). These genes are frequently upregulated in resistant strains. Previous studies have shown higher expression of CDR1, CDR2, and MDR1 in fluconazole-resistant isolates (Lee et al., 2021) (Liu and Myers, 2017). In particular, CDR1 and CDR2 play important roles in the early stages of biofilm-associated azole resistance, with CDR1 expression significantly increased during biofilm formation (Mukherjee et al., 2003; Shi et al., 2019).

In the multivariate logistic regression analysis, biofilm-forming ability showed a positive association with RVVC risk, although the result was only marginally significant. This suggests that stronger biofilm formation may independently contribute to disease recurrence. The lack of statistical significance could be partly due to the limited sample size, which reduced the power to detect modest effects. Nevertheless, the consistent trend across both univariate and multivariate analyses supports the role of biofilm formation in antifungal resistance and RVVC recurrence. Future studies with larger cohorts are warranted to validate these findings.

Previous research has confirmed that biofilm-associated resistance in C. albicans is related to high expression of efflux pump genes. This study further verified the correlation between efflux pump expression and biofilm-associated resistance. No prior reports have specifically linked efflux pump genes to biofilm-associated resistance in RVVC-causing Candida strains. In this study, isolates were grouped by biofilm-forming ability to determine whether CDR1, CDR2, and MDR1 expression correlated with biofilm strength.

Our findings show that after biofilm formation, the expression levels of CDR1, CDR2, and MDR1 all tended to increase, with CDR1 and CDR2 significantly upregulated. In particular, CDR1 and CDR2 expression was higher in RVVC strongly positive strains than in all other groups, although not significantly different from RVVC moderately positive strains. MDR1 expression showed no significant differences among groups, suggesting that biofilm formation may not affect its expression. The expression of CDR1 and CDR2 was positively correlated with biofilm-forming ability, especially in RVVC strains. This suggests that increased expression of these efflux pump genes may contribute to stronger biofilm formation and higher recurrence, confirming for the first time that efflux pumps are part of the resistance mechanism in RVVC-associated C. albicans strains (Pan et al., 2023; Rodrigues et al., 2023).

CDR1 and CDR2 are involved in biofilm formation and play an important role in enhancing drug resistance. These findings are consistent with earlier studies showing higher expression of CDR1, CDR2, and MDR1 in fluconazole-resistant strains (Lohse et al., 2018). Induction of efflux pump expression involves multiple co-activators, and transcriptional activation of MDR1 is a common route to fluconazole resistance. This study confirms that CDR1, CDR2, and MDR1 are key contributors to antifungal resistance in C. albicans. Although MDR1 expression did not change significantly before and after biofilm formation, this may be due to limited sample size. Future studies should increase sample size and experimental replicates to further investigate differential expression of resistance genes across biofilm groups (Tekintaş et al., 2020). Although MDR1 expression showed a relative increasing trend after biofilm formation in all groups, this difference was not statistically significant, which is contrary to most previous reports (Brand et al., 2021). Several factors may account for this result. First, these *Candida albicans* strains may rely more on the functions of CDR1 and CDR2 in biofilm-associated resistance, thereby showing significant upregulation of these two genes (Kovács et al., 2019). Second, the involvement of MDR1 in biofilm formation may be limited, or MDR1 may only play an auxiliary role (36). We also speculate that the upregulation of MDR1 might occur predominantly during the early stage of biofilm formation (e.g., within 2 hours), whereas its expression does not remain elevated as the biofilm continues to mature.

Our study focused exclusively on *Candida albicans*. In future investigations, non-*albicans Candida* species will be included to allow a more comprehensive and in-depth exploration. We collected clinical isolates from patients with VVC and RVVC, assessed their biofilm-forming capacity, and compared antifungal susceptibility and resistance gene expression before and after biofilm formation. We found that biofilm formation significantly increased the MIC values of certain antifungal agents. In RVVC isolates, the resistance genes CDR1 and CDR2 were markedly upregulated. However, the present study has some limitations: the number of isolates was relatively small, and *in vivo* validation experiments were lacking. These limitations will be addressed in future work.

Importantly, our findings indicate that within the VVC group, stronger biofilm formation was associated with higher MBEC values. In the RVVC group, clotrimazole consistently demonstrated lower MIC and MBEC values, supporting its use as a first-line treatment for vulvovaginal candidiasis. Furthermore, CDR1 and CDR2 expression levels were significantly higher in strongly biofilm-forming RVVC strains compared with other groups. Taken together, these results suggest that biofilm formation is one of the mechanisms underlying reduced antifungal susceptibility and upregulation of efflux pump genes in *C. albicans*, a phenomenon particularly pronounced in RVVC.

Therefore, individualized treatment strategies and rational antifungal use are essential to prevent drug misuse. Targeting biofilm formation represents a novel therapeutic avenue for *C. albicans* infections. In recent years, several drugs originally developed for other diseases have been reported to exhibit antifungal activity, either alone or in combination with conventional antifungal agents. Such approaches may enhance the susceptibility of *C. albicans* and offer new possibilities for the treatment of VVC and RVVC.

In conclusion, biofilm formation is closely related to antifungal resistance in C. albicans. Reducing efflux pump gene expression and inhibiting biofilm development may help lower resistance. Personalized treatment regimens and rational antifungal use are essential to prevent misuse. Combination therapies, targeting fungal virulence factors, and modulating host immunity are promising strategies. Anti-biofilm agents offer a new approach for treating Candida infections. In recent years, various drugs traditionally used for other diseases have demonstrated antifungal activity either alone or in combination with conventional agents (Lass-Flörl et al., 2001; Kovács et al., 2019; Tits et al., 2020; Brand et al., 2021), offering new possibilities for treating VVC and RVVC. Further research is needed to explore the mechanisms of action of these agents and confirm their efficacy through *in vitro* and *in vivo* studies.

## 6 Conclusion

The study demonstrates that biofilm formation plays a crucial role in the antifungal resistance of *Candida albicans*, particularly in recurrent vulvovaginal candidiasis (RVVC). A higher proportion of strong biofilm-forming strains was observed in the RVVC group compared to the VVC group, and these strains exhibited significantly elevated MIC and MBEC values across multiple antifungal agents. Notably, stronger biofilm formation was associated with increased expression of drug efflux pump genes, especially CDR1 and CDR2, suggesting a mechanistic link between biofilm development and enhanced resistance. Among the antifungal agents tested, clotrimazole consistently showed the lowest MIC and MBEC values in the RVVC group, indicating its potential as a preferred first-line treatment. Overall, biofilm formation contributes to reduced antifungal susceptibility and upregulation of resistance genes in *Candida albicans*, with a more pronounced effect in RVVC, underscoring the need for targeted therapeutic strategies in managing biofilm-associated infections.

## Data Availability

The original contributions presented in the study are included in the article/supplementary material. Further inquiries can be directed to the corresponding author.
